# First person – Sophie Frampton

**DOI:** 10.1242/bio.059463

**Published:** 2022-06-15

## Abstract

First Person is a series of interviews with the first authors of a selection of papers published in Biology Open, helping early-career researchers promote themselves alongside their papers. Sophie Frampton is first author on ‘ Modelling the structure of Short Gastrulation and generation of a toolkit for studying its function in *Drosophila*’, published in BiO. Sophie is a postdoc in the lab of Hilary Ashe at The University of Manchester, UK, investigating the processes governing tissue patterning and mechanisms of cell signal regulation.


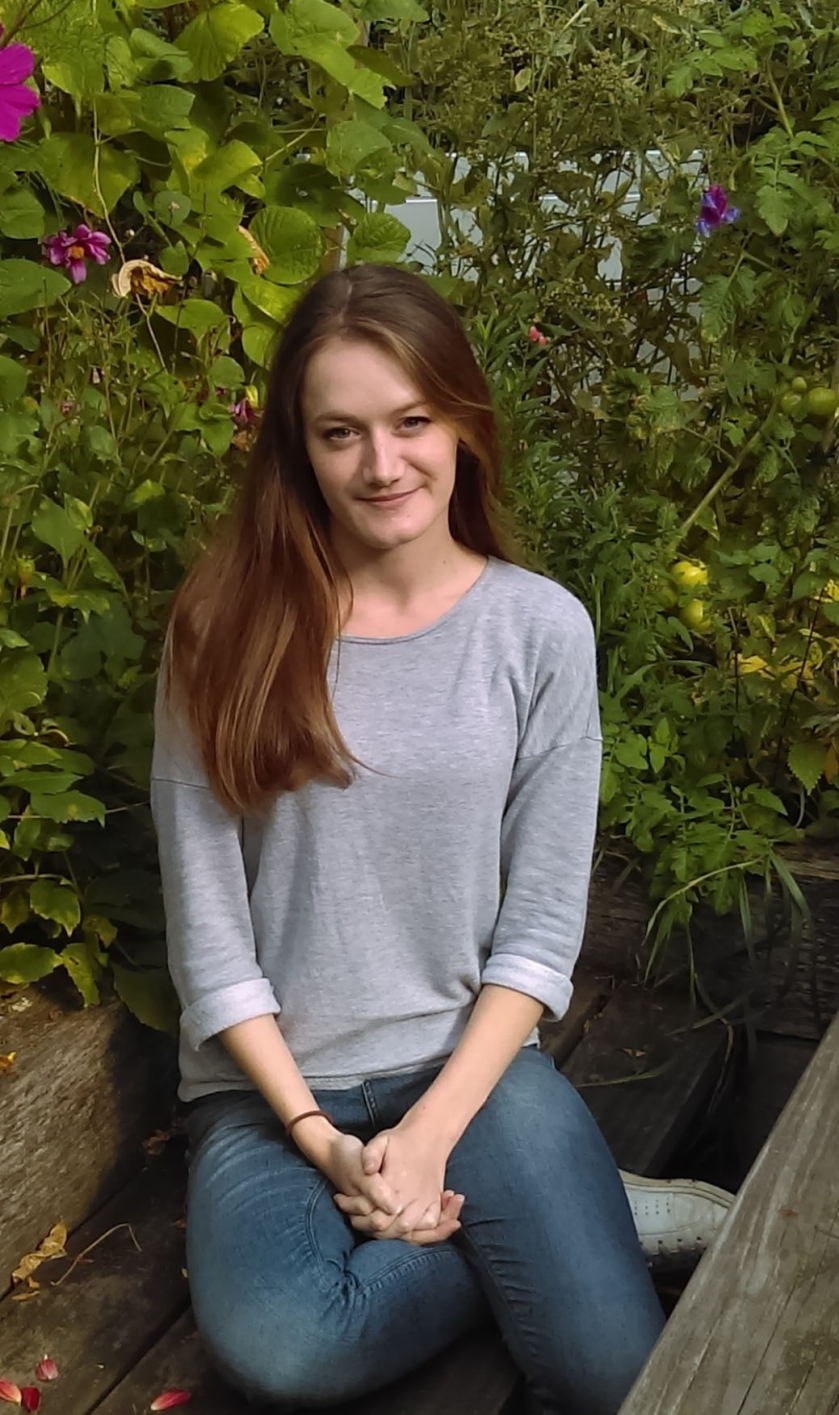


**Sophie Frampton**

**What is your scientific background and the general focus of your lab?**

I studied Biological Sciences at Oxford University before moving to Manchester to pursue my master's in developmental biology. In 2016 I began my PhD as a joint student in the labs of Hilary Ashe and Clair Baldock at The University of Manchester. My project made use of biophysical, genetic, and developmental techniques to investigate the role of Short Gastrulation (Sog) in bone morphogenetic protein (BMP) signal regulation during *Drosophila* dorsal-ventral patterning. Following the completion of my PhD in 2021, I gained a postdoc position in the Ashe lab to expand on work from my PhD.

The Ashe lab uses the *Drosophila* embryo and ovary to investigate a variety of fundamental biology questions. Work in collaboration with the Baldock lab focusses on mechanisms of BMP signal regulation in the form of interdisciplinary structural/developmental biology projects. The Ashe lab also studies how signalling filopodia receive and modulate the BMP signal in the ovary. Other research themes in the Ashe lab include the regulation of gene expression dynamics via transcription, mRNA stability, and translation during *Drosophila* embryogenesis. This is achieved through a combination of high-resolution imaging techniques and mathematical modelling.

**How would you explain the main findings of your paper to non-scientific family and friends?**

Mechanisms of cell communication via signalling proteins during embryo development and adult life are essential for the development and maintenance of healthy tissues. Here we focus on how activity levels of a particular signalling molecule (BMP), which is needed to define the back-to-front axis of an embryo, are controlled by a protein called Sog. To enable testing of different *sog* mutants in fruit fly embryos, we use genetic engineering to create a fly line in which we can easily express different mutant versions of *sog*. We make use of this system to test the requirement of a fatty-acid protein modification for Sog function and find that this modification contributes only a small amount to Sog-mediated BMP regulation. We next made use of electron microscopy (EM) to image Sog and construct a 3D model of the protein. We find that the EM Sog model is consistent with a Sog structure predicted by the artificial intelligence program AlphaFold. From this analysis we also identify features of Sog that may contribute to its activity and explain functional differences between Sog and its human equivalent, Chordin.

**What are the potential implications of these results for your field of research?**

We have generated a new *sog* mutant *Drosophila* line in which the *sog* start codon is replaced with a recombination sequence. This new mutant line permits expression of *sog* CDS variants at the endogenous *sog* locus. In addition, we have used negative stain EM to create a 3D model of Sog. With this EM model, and in combination with the AlphaFold predicted Sog structure, we infer features of Sog function based on structural characteristics. Ultimately, our unique approach to the analysis of Sog has developed important tools that will facilitate future research and the molecular dissection of extracellular BMP regulation.

**Figure:**
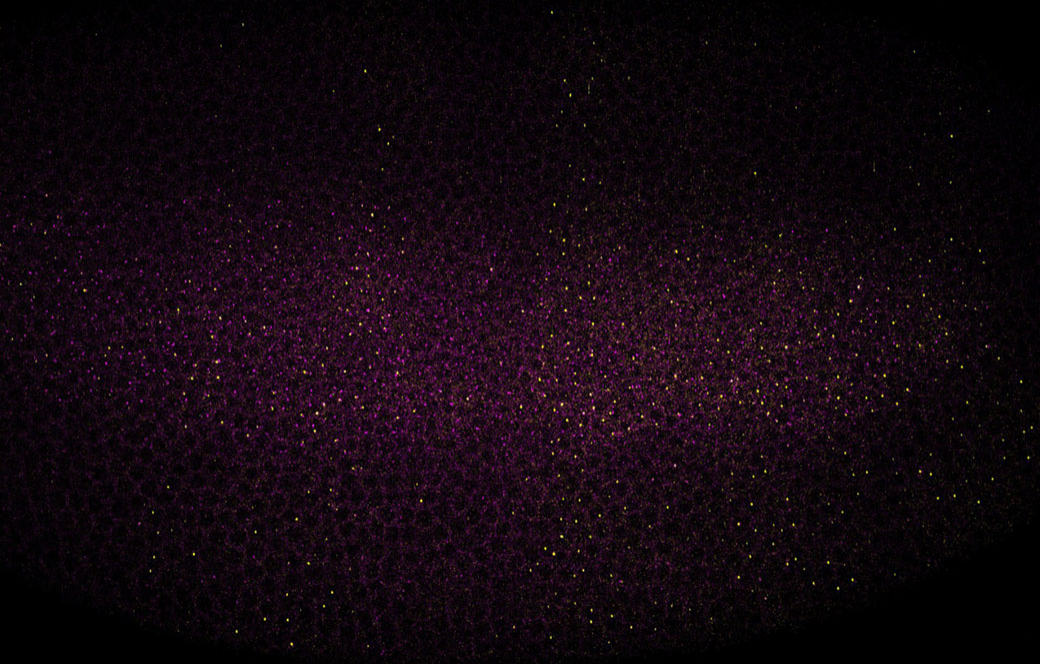
***short gastrulation* (*sog*) and *fushi tarazu* (*ftz*)*-lacZ* expression in an early *Drosophila* embryo.** Individual *sog* mRNAs and transcription sites (magenta) are visualised by single molecule fluorescent RNA *in situ* hybridisation. This embryo also expresses a *ftz-lacZ* reporter (yellow).

**What has surprised you the most while conducting your research?**

I have been surprised by how long it can take to find an embryo of the right age and orientation on a slide to image!

**What, in your opinion, are some of the greatest achievements in your field and how has this influenced your research?**

The development of high resolution and quantitative imaging techniques, as well as analysis pipelines, for both live and fixed *Drosophila* embryos have enabled fundamental biological processes to be analysed *in vivo* and in fine detail. For instance, in our research these methods have allowed us to detect subtle changes to BMP signalling. In the field of structural biology, the release of AlphaFold in 2021 has revealed information about protein structures that may be difficult to solve via experimental methods due to the properties of the protein of interest.

**What's next for you?**

In my current role at the Ashe lab I continue to investigate Sog function in *Drosophila*. I am, however, looking for a new postdoc position in the field of developmental biology.
